# Prevalence of latent tuberculosis infection in healthcare workers in tertiary care hospitals of Pakistan

**DOI:** 10.12669/pjms.36.2.936

**Published:** 2020

**Authors:** Rabia Sadaf, Tehmina Munir, Sheroze Farrukh, Saleem Abbasi

**Affiliations:** 1 Rabia Sadaf, Postgraduate Trainee, Department of Microbiology, Army Medical College, Rawalpindi, Pakistan; 2 Tehmina Munir, Professor, Department of Microbiology, Hitech IMS, Taxila, Pakistan; 3 Sheroze Farrukh, M. Phil, (Microbiology), National University of Medical Sciences, Rawalpindi, Pakistan; 4 Saleem Abbasi, Research Officer, Department of Medical Education, Rawal Institute of Health Sciences, Islamabad, Pakistan

**Keywords:** Healthcare workers, IGRA test, Latent TB infection, Tuberculosis

## Abstract

**Objective:**

To determine the prevalence of latent tuberculosis infection (LTBI) in healthcare workers in tertiary care hospitals of Rawalpindi, using interferon gamma release assay.

**Methods:**

It was a cross-sectional study. The samples were collected from pulmonology and microbiology departments of three hospitals; i) Military Hospital, Rawalpindi, ii) Fauji Foundation Hospital, Rawalpindi and iii) Pakistan Institute of Medical Sciences, Islamabad. The study was completed in one year from January 2017 to January 2018. Fifty-five asymptomatic healthcare workers of both genders between the ages of 18-50 years with a working tenure of at least one year in concerned departments were included and those with active tuberculosis were excluded from the study. Whole blood from subjects was collected and plasma was checked for interferon gamma value by IGRA (Interferon gamma release assay).

**Results:**

In this study of total 55 healthcare workers a high prevalence 22 (40.0%) of latent tuberculosis was found. When LTBI distribution was analyzed within occupational categories, the most frequently affected were sanitary workers 3 (100.0%), nurses 5 (50.0%), doctors 6 (43%) and nursing assistants 2 (40%).

**Conclusion:**

The prevalence of LTBI in healthcare workers is alarmingly high in our local healthcare settings.

## INTRODUCTION

Tuberculosis (TB) continues to be a major health problem worldwide. About 1.7 million people die of tuberculosis every year. Quite remarkably, the current incidence of tuberculosis is at an all-time high (>9 million). Eighty percent of the active cases come from 22 countries with low socioeconomic status.^1^ In terms of incidence of tuberculosis, the WHO African region is on top with 356 cases/100,000 populations per year. On the other hand, high prevalence is seen in densely populated Asian countries (Bangladesh, China, India, Indonesia and Pakistan).^2^ Along with WHO’s Eastern Mediterranean Region countries (EMR) like Afghanistan, Egypt, Iraq, Morocco, Somalia, Sudan and Yemen, Pakistan is currently rated ‘D’ which means that disease burden and mortality is very high. Pakistan carries about two-thirds of the total disease burden of all the 22 EMR countries, an alarming proportion indeed.^3^ One of the many important challenges in the control of tuberculosis is the early detection and management of LTBI. About 10% of individuals infected with *Mycobacterium tuberculosis* develop active disease whereas the remaining 90%manage to suppress the bacterial invasion.^4^

The global effort of controlling TB is spearheaded by World Health Organization’s “end TB strategy” which aims to lower the incidence of TB by 90% and TB deaths by 95% by 2035. Studies have determined that in order to achieve these targets, a multipronged strategy is required; on one hand to detect and treat active TB cases and on the other to reduce the reservoir of latent TB cases.^5^

Healthcare workers (HCWs) in general are considered a high-risk group for LTBI.^4^ A study done in a TB hospital in New Delhi using DNA fingerprinting in Mycobacterial isolates from patients revealed that most of the affected healthcare workers had hospital-acquired primary pleural tuberculosis rather than reactivation of previously acquired tuberculosis. The risk of HCWs developing tuberculosis varies with the nature of their duties. HCWs working in microbiology laboratories, TB wards and emergency rooms are at highest risk than those working elsewhere.^6^ Contributing factors to this propensity include increased exposure, poor infection control protocols, improper respiratory hygiene and bad cough etiquette.^7^ Among laboratory workers, those involved in collection, induction and processing of sputum have a very high risk of exposure and transmission. Similarly, workers involved in bronchoscopy, endotracheal intubation and disposal of biological wastes are also considered high risk.^6^ A recent meta-analysis proved that healthcare workers were at three-fold risk to develop tuberculosis as compared to general population. No other population subgroup is more highly exposed to multi-drug resistant tuberculosis (MDR) than healthcare workers and thus, have six-fold risk to contact MDR tuberculosis.^8^

While the significance of LTBI detection cannot be overemphasized, there is no gold standard test for its diagnosis. The quantity of *Mycobacterium tuberculosis* in LTBI patients is minimal. Consequently, LTBI is detected by measuring the immune reactions of the host in response to *Mycobacterium tuberculosis*. Two widely used screening tests are the conventional Tuberculin skin test (TST) and the more modern Interferon gamma release assays (IGRAs).^9^ Conventionally TST has been in use for many decades but it has several limitations like personal bias, late results, two patient visits 72 hours apart, low sensitivity, cross-reactivity with previous BCG vaccination and atypical Mycobacteria as well as false-negative results in immunosuppressive states.^10^ IGRA was received with great enthusiasm mainly due to confounding factors that limit the reliability of TST. After stimulation with *Mycobacterium tuberculosis* antigens, the sensitized T cells release gamma interferon which is accurately measured by IGRA. In contrast to TST, IGRA obviates the requirement of multiple visits and eliminates personal bias.^6^ However, a high cost is an important hurdle in low socioeconomic countries. Moreover, the availability of specific laboratory requirements and trained laboratory staff are other issues. We designed a study to find out the prevalence of latent tuberculosis infection in healthcare workers working in tertiary care hospitals of a large city in Pakistan using QuantiFERON TB Gold plus, a more recent four tube version of IGRA.

## METHODS

It was a cross-sectional study, carried out at the department of microbiology, Army Medical College and in collaboration with Armed Forces Institute of Pathology, Rawalpindi, NUMS affiliated with the Military Hospital, Rawalpindi, it is 1100 bedded tertiary care facility. The samples were collected from the pulmonology department of Military Hospital, Rawalpindi as well as from the pulmonology department of Pakistan Institute of Medical Sciences (PIMS) and microbiology department of Fauji Foundation Hospital. The study was completed in one year from January 2017 to January 2018.

The healthcare workers of both genders between the ages of 18-50 years with at least one-year work tenure in the pulmonology or microbiology departments were screened for enrollment. These were asymptomatic individuals with a normal chest radiograph and normal ESR. The healthcare workers with a suspicion or confirmation of active tuberculosis and those suffering from other chronic co-morbidity (ESRD, cardiovascular disease) were excluded. Permission from the institutional ethical committee was obtained and informed written consent was administered. Data was collected regarding age, gender, occupational subgroups, duration of exposure, BCG vaccination status and previous medical history.

A whole blood sample of 4 ml was collected in special blood collecting tubes containing TB peptide antigens (ESAT-6 and CFP-10) and transported to Armed Forces Institute of Pathology. After incubation for 24 hours, all tubes were centrifuged for 15 minutes and plasma was separated. These plasma samples were added to a microtiter ELISA plate and standardized guide of the manufacturer was applied. Then the optical density of each well was measured within 5 minutes using an ELISA reader. Results were analyzed by using QFT-Plus analysis software. The test was considered positive for an interferon gamma response to either TB Antigen tube that was significantly above the ‘nil’ interferon gamma (IFN-ɤ IU/ml) value. The plasma sample from mitogen tube served as a positive control. The cut-off value for a positive test result was 0.35 IU/ml of interferon gamma in the plasma after stimulation. All the positive results were screened to rule out active TB by doing their chest X-ray and evaluation was done by a physician for symptoms of active TB.

### Statistical analysis

The systematically calculated study sample was 55 cases using WHO calculator with the assumption of 90% LTBI rate from a previous study, and using 95% confidence level and 5% alpha error. Data were analyzed using SPSS Version 21. Frequency and percentages were calculated for qualitative variables whereas the quantitative data were expressed as mean ± standard deviation.

## RESULTS

In this study male gender was in majority with 33 (60%) cases. The most represented occupational subgroup was laboratory technicians, doctors and then nurses with the mean age of 37.5 ± 10.8 years. Most of the HCWs 43(78.1%) gave a history of less than 10 years’duration of exposure. Positive vaccination status was common, as 41 (74.5%) HCWs had BCG vaccination scar. Most of the participants 50(91%) had no exposure to any TB patient in close relation. The majority belonged to middle class 38(69.1%). The overall prevalence of latent tuberculosis infection in the study population was 22(40%). These cases showed positive IGRA test findings for the detection of LTBI. Furthermore, the socio-demographic and clinical features of participants were compared according to IGRA results. The most frequently affected occupations were sanitary workers 3 (100.0%), nurses 5 (50.0%), doctors 6 (43%) and nursing assistants 2 (40%). The proportionate analysis revealed that female gender (50%), low socioeconomic status (56%), absence of BCG scar (50%) and exposure duration of <10 years (46.5%) were more likely to have LTBI. (Table-I)

**Table-I:** Association of socio-demographic and clinical features with IGRA findings (n=55).

	IGRA positive (n=22)	IGRA negative (n=33)	p-value
***Age (years)***			
20-30 (n=20, 36.4%)	7 (35%)	13 (65%)	0.62
31-40 (n=16, 29.1%)	8 (50%)	8 (50%)	
41 or above (n=19, 34.5%)	7 (36.8%)	12 (63.2%)	
***Gender***			
Male (n=33, 60.0%)	11 (33.3%)	22 (66.7%)	0.21
Female (n=22, 40.0%)	11 (50%)	11 (50%)	
***Occupational Subgroups***			
Doctors (n=14, 25.5%)	6 (43%)	8 (57%)	0.14
Nurses (n=10, 18.2%)	5 (50%)	5 (50%)	
Laboratory Technicians (n=23, 41.8%)	6 (26%)	17 (74%)	
Ward Boys (n=5, 9.1%)	2 (40%)	3 (60%)	
Sanitary Workers (n=3, 5.5%)	3 (100%)	0 (0.0%)	
***Presence of BCG Scar***			
Yes (n=41, 74.5%)	15 (36.6%)	26 (63.4%)	
No (n=14, 24.5%)	7 (50%)	7 (50%)	0.37
***Contact with household member with TB***			
Yes (n=5, 9.5%)	2 (40%)	3 (60%)	1.0
No (n=50, 90.5%)	20 (40%)	30 (60%)	
***Socioeconomic Status***			
Low (n=9, 16.4%)	5 (56%)	4 (44%)	0.58
Middle (n=38, 69.1%)	14 (37%)	24 (63%)	
Upper Middle (n=8, 14.5%)	3 (37.5%)	5 (62.5%)	
***Duration of Exposure (years)***			
>10 (n=12, 21.8%)	2 (17%)	10 (83%)	0.06
<10 (n=43, 78.2%)	20 (46.5%)	23 (53.5%)	

## DISCUSSION

This study highlights a significantly high proportion of healthcare workers with LTBI in tertiary care settings of Pakistan. Among the top 30 high tuberculosis burdened countries, Pakistan is placed at the 5^th^ position.^11^ Mortality from TB in Pakistan was estimated to be 26 deaths per 100,000 population.^3^ Individuals with latent tuberculosis are considered to be the most important reservoirs of the disease with a huge potential to progress to active disease. A lot of research effort is taking place worldwide to detect LTBI, however, in Pakistan, not much data is available regarding its spread, control or management. One previous effort revealed 48% LTBI in prisoners.^12^ Another study witnessed 80-90% cases of LTBI in children under 5 years of age having close contact with a TB patient.^13^ No local data on HCWs is available at present.

The 40% prevalence of LTBI in healthcare workers is an alarming situation for the National TB program in Pakistan. Studies done worldwide, especially in low and middle-income countries showed a more or less similar picture. A study done in rural India on HCW, found LTBI in 40% cases by IGRA and 41% by Tuberculin skin test (TST).^14^ China was second to India in comparison to the incidence of tuberculosis along with 1.3 million new cases of TB patients per year including a high percentage of MDR-TB patients. According to Zhang et al., 2013,the prevalence of LTBI was calculated as 37.4% in high-risk areas by IGRA.^15^ A meta-analysis on the prevalence of LTBI among HCW showed pooled prevalence of 47%, with 54% from Bangladesh, 43% in India and 54% from China.^7^ The moderate and low burden countries had a comparatively lower prevalence of LTBI than our study. Another study from Al-jazeera state in Sudan reported 35.7% LTBI in HCWs.^16^ A study from Portugal demonstrated LTBI prevalence of 32.6% in HCW.^17^ African region is a high burden geographical area in which significant size of population has tuberculosis, South Africa has a LTBI prevalence of 64% in its population, whereas a pooled analysis showed overall LTBI rates of up to 57% in HCWs.^7^ Another study was done in Taiwan, the prevalence of latent TB in HCW was found to be 14.5% by IGRA.^18^ A study from Rome, Italy witnessed low LTBI burden 16.1% in a teaching hospital.^8^ Despite being a high-risk group, the exposure of healthcare workers to TB in Pakistan has not been studied earlier which is one of the advantages of the present study. Furthermore, we have highlighted the risk of occupational exposure of HCWs to tuberculosis infection. We also utilized the modern diagnostic technique of IGRA which eliminates the limitations of conventional tuberculin skin test.

The present study was of a cross-sectional nature with a time period of one year and study participants were healthcare workers of tertiary care hospitals working in only one large city of Pakistan. A cohort study with a larger sample size and extended duration and spectrum needs to be done in order to address these limitations.

## CONCLUSION

By using IGRA, we found that 40% of the study subjects have latent tuberculosis. This shows that healthcare workers are at an increased risk to develop latent tuberculosis infection due to a high level of occupational exposure to patients with active disease. There is a need to educate and advocate healthcare workers regarding applying proper preventive protocols for prevention of TB.

### Author`s Contribution:

**RS** conceptualized and conducted data collection and wrote initial draft of manuscript, is responsible for authenticity of data.

**TM and SF** helped in manuscript writing and reviewed final draft.

**SA** conducted statistical analysis, interpretation and critical revision of manuscript.
